# Targeting IFNGR/IL6R or downstream JAK1/JAK2 to control GvHD

**DOI:** 10.18632/oncotarget.26291

**Published:** 2018-11-06

**Authors:** Kidist Ashami, John F. DiPersio, Jaebok Choi

**Affiliations:** Jaebok Choi: Division of Oncology, Department of Medicine, Washington University School of Medicine, St. Louis, MO, USA

**Keywords:** GvHD, IFNGR, IL6R, JAK1/JAK2, baricitinib

Allogeneic hematopoietic stem cell transplantation (allo-HSCT) is the most effective treatment for various hematological malignancies such as refractory or relapsed leukemia and marrow failure states. The therapeutic benefits of allo-HSCT are mainly derived from an anti-tumor or graft-versus-leukemia (GvL) effect that is mediated by allo-reactive T cells in the donor graft. However, the same donor T cells could also cause graft-versus-host disease (GvHD) by recognizing not only recipient leukemic cells but also healthy tissues and organs as foreign and damage them. Current treatments of GvHD mainly focus on the immunosuppression of the donor T cells via pharmacologic prophylaxis or donor T cell depletion [1]. Given the tight link between GvL and GvHD, this type of treatment often diminishes the therapeutic efficacies of allo-HSCT thereby increasing the risk of leukemia relapse and subsequent mortality [2]. Hence, the need for treatment options that are less systemically immunosuppressive and could also provide durable prophylactic and curative responses without compromising GvL effects cannot be over-emphasized. To this effect, identifying optimal molecular targets is critical to develop therapeutic strategies to selectively overcome GvHD while boosting the beneficial GvL effects.

Accordingly, we have shown that the genetic deletion of interferon gamma receptor (IFNGR) or its downstream chemokine receptor CXCR3 in donor T cells results in donor T cell trafficking away from GvHD organs, thus decreasing the severity of GvHD while maintaining robust engraftment and GvL [3]. Since IFNGR signaling is mediated by both JAK1 and JAK2, we then hypothesized that an *in vivo* administration of JAK1/JAK2-specific inhibitors should significantly reduce GvHD while preserving GvL. As we previously reported in 2012, our group was the first to demonstrate that the administration of ruxolitinib (RUX), a JAK1/JAK2 inhibitor, to MHC-mismatched murine allo-HSCT recipients phenocopies the reduced GvHD potential of IFNGR KO T cells [3]. Subsequently, we have reported that RUX maintains the GvL effects in our preclinical models of allo-HSCT [4]. Of note, mice transplanted with IFNGR KO T cells in combination with RUX resulted in 100% overall survival while only a ∼50-80% survival was observed in the recipient mice transplanted with IFNGR KO T cells alone [4]. Thus, we speculated that RUX must inhibit other non-IFNGR signaling pathways which are themselves dependent on JAK1/JAK2.

**Figure 1:**
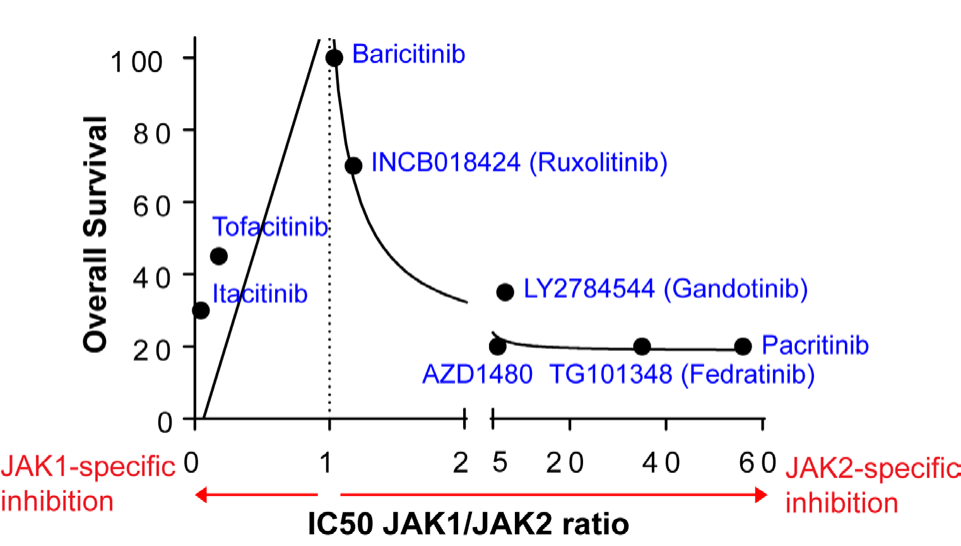
The effect of JAK inhibitors on GvHD is demonstrated by overall survival of allo-HSCT recipient mice (B6 to Balb/c) An *in vivo* administration of JAK1- or JAK2-specific inhibitors reduces GvHD. However, BARI and RUX, balanced JAK1/JAK2 inhibitors, are the most potent in GvHD prevention. The mean overall survival of vehicle control groups is 5%.

Another major cytokine receptor signaling mediated by JAK1/JAK2 is the interleukin-6 receptor (IL6R) signaling. Therefore, we hypothesized that blocking both IFNGR and IL6R signaling would result in complete prevention of GvHD while potentially preserving GvL. We have demonstrated that genetic deletion of IFNGR in donor T cells plus the *in vivo* administration of anti-IL6R antibody to recipient mice are sufficient to completely prevent GvHD [5]. In addition, pharmacologic inhibition of IFNGR/IL6R signaling with another JAK1/JAK2 inhibitor, baricitinib (BARI), completely prevents GvHD while enhancing the GvL effects [5]. Our data, therefore, strongly demonstrate that the optimal targets for GvHD are IFNGR and IL6R. These results provide important insights into optimal JAKs to target for the prevention and treatment of GvHD without abrogating GvL. While comprised of four different non-receptor kinases (JAK1, JAK2, JAK3 and TYK2), the first three members of the JAK families have been reported to be ideal targets for GvHD treatments due to their ability to regulate the immune cells that underlie GvHD [6]. Our data, however, strongly suggest that balanced inhibition of JAK1/JAK2, while preserving JAK3 that is necessary for regulatory T cells (see below), is critical for complete eradication of GvHD [3-5] (Figure 1). Although JAK1- or JAK2-specific inhibitors such as tofacitinib and gandotinib can reduce GvHD in preclinical murine allo-HSCT models, they are not as effective as RUX and BARI, balanced JAK1/JAK2 inhibitors. This may be due to the fact that IFNGR and IL6R signaling (which require both JAK1/JAK2 for signal transduction) are stochastically inhibited most by RUX and BARI comparing to other cytokine signaling pathways that are dependent on either JAK1 or JAK2 alone. Likewise, BARI, the most balanced JAK1/JAK2 inhibitor, is significantly more potent in preventing GvHD than RUX [5] in our MHC-mismatched mouse allo-HSCT models (Figure 1).

Given the similarity in structures and JAK inhibition profiles of RUX and BARI, understanding the mechanisms underlying the superiority of BARI to RUX will further help guide the rational design of future randomized clinical trials to validate the potential benefit of JAK1/JAK2 inhibitors for the prevention and treatment of GvHD. While both BARI and RUX equally inhibit GvHD-inducing Th1/Th2 cell differentiation and CXCR3 expression, BARI’s superiority to RUX in prevention of GvHD is partially due to a robust upregulation of regulatory T cells by preserving IL2R-JAK1/JAK3-STAT5, and reduction of antigen presenting cells’ allo-reactivity [5]. In addition, differences in pharmacokinetics between BARI and RUX could also explain the superiority of BARI to RUX [7, 8]. Furthermore, we are currently performing RNA profiling analyses, adaptive kinome reprogramming analyses, and off-target kinase analyses to identify genes and kinases that are differentially regulated by BARI vs RUX.

In addition to its potent role in GvHD prophylaxis, our data also show that BARI treats ongoing GvHD effectively [5]. Thus, it is possible that BARI not only modulates immune cell functions as described above, but also promotes restoration of GvHD damaged tissues. Supporting our hypothesis, we have found that both RUX and BARI enhance human intestinal organoid growth (unpublished data). Hence, further research will be required to identify the genes and pathways that mediate BARI-induced recovery of damaged tissues due to GvHD.

Our data also show that BARI preserves and even enhances GvL effects [5]. This effect could be a result of downregulation of PD-L1 on leukemic cells [5]. Nonetheless, the effect of BARI on GvL was examined only in one single preclinical model (B6 to Balb/c along with A20 leukemic cells). Thus, a further investigation using different leukemia models is required to precisely determine the effect of BARI on GvL.

Although BARI completely prevents and treats GvHD while enhancing the GvL effects in allo-HSCT models, it can inhibit other cytokine/growth factor receptor signaling pathways that are mediated by JAK1 or JAK2, which might result in undesired side effects. Thus, these potential side effects call for the development of more targeted approaches that specifically block IFNGR and IL6R.
